# Trifunctional Graphene Quantum Dot@LDH Integrated Nanoprobes for Visualization Therapy of Gastric Cancer

**DOI:** 10.1002/adhm.202100512

**Published:** 2021-06-10

**Authors:** Bin Wu, Kun Li, Feiyue Sun, Jintong Niu, Rongrong Zhu, Yechang Qian, Shilong Wang

**Affiliations:** ^1^ Research Center for Translational Medicine at East Hospital Tongji University School of Life Science and Technology Shanghai 200092 China; ^2^ Department of Respiratory Disease Baoshan District Hospital of Integrated Traditional Chinese and Western Medicine Shanghai 201900 China

**Keywords:** gastric cancer, graphene quantum dots, layered double hydroxide, nanoprobes, visualization therapy

## Abstract

Visualization technology has become a trend in tumor therapy in recent years. The superior optical properties of graphene quantum dots (GQDs) make them suitable candidates for tumor diagnosis, but their tumor targeting and drug‐carrying capacities are still not ideal for treatment. Sulfur‐doped graphene quantum dots (SGQDs) with stable fluorescence are prepared in a previous study. A reliable strategy by associating layered double hydroxides (LDHs) and etoposide (VP16) is designed for precise visualization therapy. Trifunctional LDH@SGQD‐VP16 integrated nanoprobes can simultaneously achieve targeted aggregation, fluorescence visualization, and chemotherapy. LDH@SGQD‐VP16 can accumulate in the tumor microenvironment, owing to pH‐sensitive properties and long‐term photostability in vivo, which can provide a basis for cancer targeting, real‐time imaging, and effect tracking. The enhanced therapeutic and attenuated side effects of VP16 are demonstrated, and the apoptosis caused by LDH@SGQD‐VP16 is ≈2.7 times higher than that of VP16 alone, in HGC‐27 cells. This work provides a theoretical and experimental basis for LDH@SGQD‐VP16 as a potential multifunctional agent for visualization therapy of gastric cancer.

## Graphical abstract

The trifunctional nanocomposite layered double hydroxide@sulfur‐doped graphene quantum dot‐VP16 (LDH@SGQD‐VP16) integrates fluorescent graphene quantum dots, layered double hydroxide, and antitumor drug VP16. It has the potential capability for pH‐sensitive tumor targeting, fluorescence imaging, and tumor therapy. Reliable imaging tracking and therapeutic ability in vitro and in vivo indicate that it can be a promising agent for visualization tumor therapy.

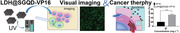

## 1 Introduction

Cancer is a complex disease that threatens human health.^[^ ^1^, ^2^, ^3^ ^]^ To combat this, effective cancer treatments, such as chemotherapy and radiation therapy, were developed after extensive research. However, both chemotherapy and radiation therapy have significant adverse effects on the human body. In recent years, nanotechnology has provided a new perspective for cancer treatment.^[^ ^4^, ^5^ ^]^ With the development of nanomolecular imaging, the difficulty of diagnosis was also gradually reduced. However, these studies are often conducted separately for diagnosis and treatment.^[^ ^6^, ^7^, ^8^, ^9^ ^]^ The feedback of treatment effect is not timely, leading to the delay of the disease. Thus, an integrated approach of visualization and precise treatment has become a significant trend in tumor treatment.^[^ ^10^, ^11^ ^]^ Multifunctional nanomaterials have become an ideal solution, owing to their multidimensional characteristics.^[^ ^12^, ^13^ ^]^ Different diagnostic media such as magnetic imaging, nuclear imaging, optical imaging, and therapeutic agents can be loaded in one package, and targeted delivery into specific sites can be achieved.^[^ ^14^, ^15^, ^16^, ^17^, ^18^ ^]^ Another advantage is that the distribution and efficacy of nanomaterials can be observed in real time.^[^ ^19^ ^]^ Although there are many excellent studies, achieving good biocompatibility and tumor‐targeted aggregation is still one of the problems.

Layered double hydroxide (LDHs), a biocompatible and biodegradable 2D nanomaterial, has been proven to be an excellent carrier for drug delivery,^[^ ^20^, ^21^ ^]^ ATP delivery,^[^ ^22^ ^]^ and DNA delivery.^[^ ^23^, ^24^ ^]^ LDH exhibits highly sensitive acidity‐induced dissolution properties, which are conducive to pH‐responsive drug release. Due to the shape effect of the plate‐like structure, LDH can be more efficiently taken via endocytosis and can linger in lysosomes for a longer amount of time.^[^ ^25^ ^]^ Unfortunately, LDH itself does not have imaging capability. Interestingly, LDH can stably coexist with some functional nanoparticles, such as CuS dots for photodynamic therapy,^[^ ^14^ ^]^ Mn and Fe_3_O_4_ for MR imaging.^[^ ^13^, ^26^ ^]^ Owing to these advantages, LDH is ideal for constructing multifunctional nanoplatforms.

Compared with MR or CT imaging, optical imaging is usually much quicker and less expensive, and its spatial resolution is the highest.^[^ ^27^ ^]^ Quantum dots (QDs), such as CdTe,^[^ ^28^ ^]^ Ag_2_S,^[^ ^29^ ^]^ and graphene QDs, are the most important components in nano fluorescence imaging. GQDs are a type of nanomaterial with excellent biocompatibility and photostability and are widely used as fluorescent probes in biological imaging^[^ ^30^, ^31^, ^32^, ^33^, ^34^, ^35^, ^36^ ^]^ and tumor therapy.^[^ ^37^, ^38^ ^]^ GQDs alone are still difficult to target for delivery to tumor sites. Moreover, the drug loading method of electrostatic binding^[^ ^39^ ^]^ or chemical bond connection^[^ ^40^ ^]^ is generally adopted on the surface of GQDs. The disadvantage is that the drugs are exposed to the biological environment without protection, making it easy to escape to non‐target tissues during transportation, and cause toxic side effects. To overcome these difficulties, the construction of multifunctional nanomaterials is the best solution.

Recently, LDH and GQDs have been used in energy batteries,^[^ ^41^ ^]^ drug delivery,^[^ ^42^ ^]^ and sensors^[^ ^43^, ^44^ ^]^ in several studies, but only a few of these were able to simultaneously achieve biological imaging and treatment. In our previous work, sulfur‐doped GQDs (SGQDs) with good biocompatibility were successfully prepared and exhibited intense blue fluorescence,^[^ ^30^ ^]^ which is suitable for bioimaging. To integrate visualization and tumor targeting, composite LDH@SGQD was synthesized by coprecipitation. The design of an integrated probe for gastric cancer treatment was completed using the traditional chemotherapy drug etoposide (VP16). Owing to the weak acidity of the tumor microenvironment,^[^ ^45^ ^]^ LDH@SGQD‐VP16 can be aggregated more easily. The long‐term stable fluorescence of the tumor site gives it the ability of biological imaging and effect tracking. Enhanced therapeutic effects were observed in vitro and in vivo. The results of the relative characterization and biological experiments proved that LDH@SGQD‐VP16 is a good multifunctional nanocomposite for precise visualization therapy of gastric cancer and provides an experimental basis for clinical applications.

## 2 Results and Discussion

### 2.1 Synthesis and Characterization

**Figure 1:**
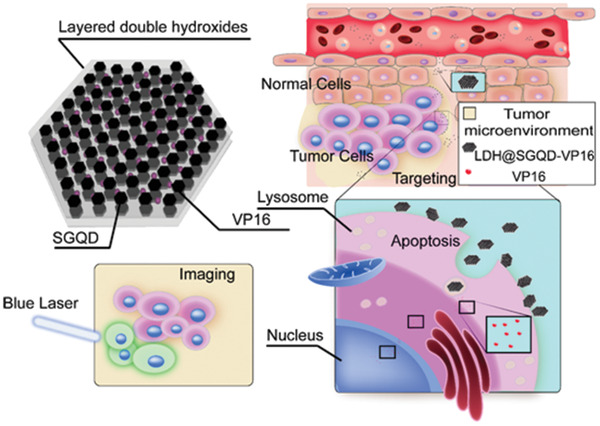
Design of trifunctional nanoprobes LDH@SGQD‐VP16 for cancer treatment. LDH@SGQD‐VP16 is composed of LDH for tumor cells targeting by pH‐sensitive properties, SGQD for fluorescence imaging, and VP16 for cancer therapy.

**Figure** **1** shows the design of the trifunctional nanoprobes LDH@SGQD‐VP16 for visual detection of cancer, tumor targeting and precise treatment. Briefly, LDH@SGQDs were synthesized using the coprecipitation method and could launch green fluorescence with a blue laser for visual imaging, and the proportion of SGQDs was ≈4.3%. In the synthesis process, drug‐loading nanocomposite system can be obtained by adding VP16 to the reaction system. The drug‐loading rate was ≈28.1%. LDH@SGQD‐VP16 is enriched by blood circulation onto the surface of tumor cells due to the acidic microenvironment, and then the nanocomplex is engulfed by the cells. VP16 is released into the cytoplasm by degradation of lysosome, causing apoptosis, for achieving cancer therapy.

**Figure 2:**
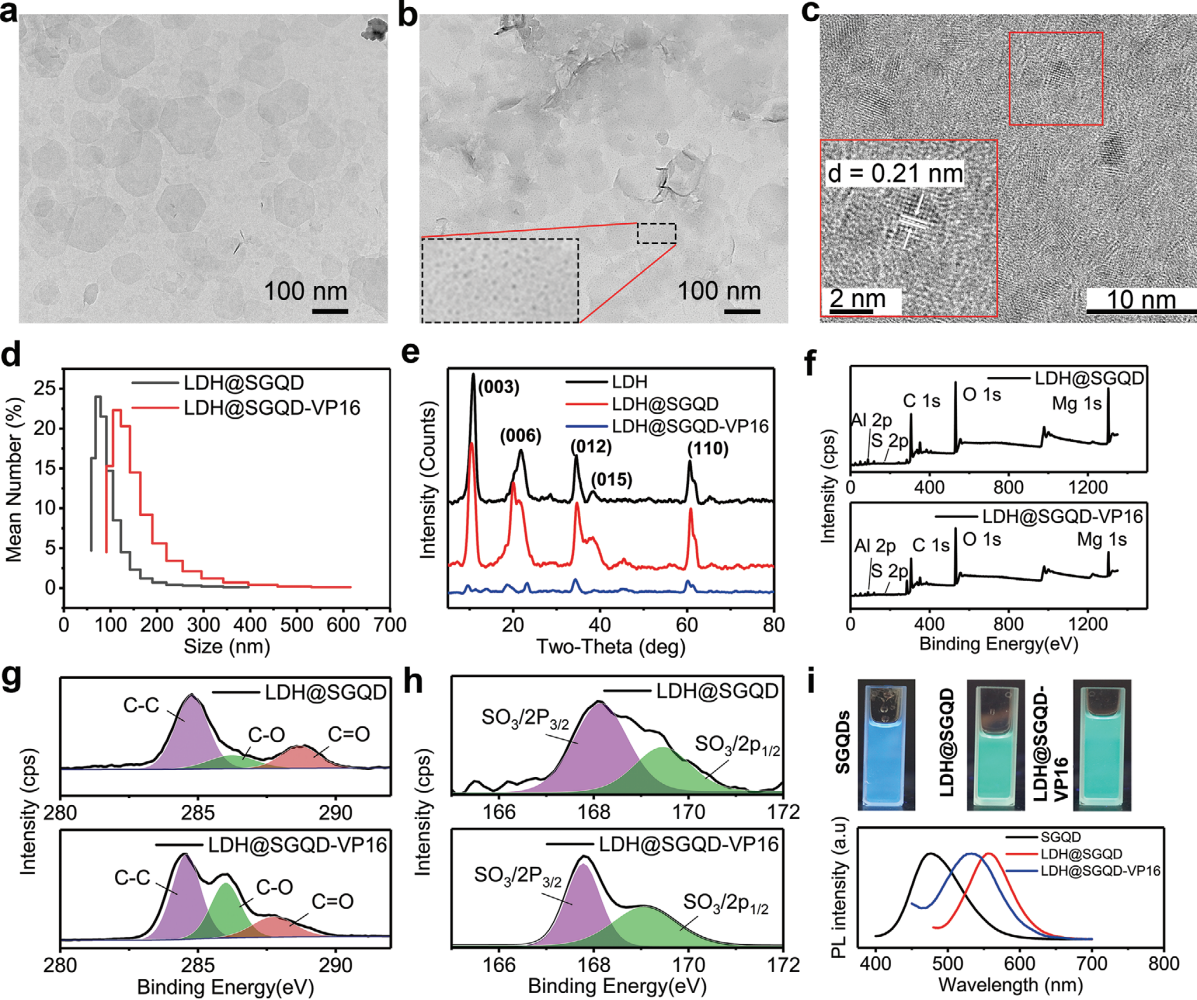
TEM images of a) LDH, b) LDH@SGQD. Inset in (b): SGQD on LDH@SGQD. HRTEM of the SGQD on c) LDH@SGQD. d) Diameter distribution of LDH@SGQD and LDH@SGQD‐VP16. e) XRD analysis of LDH, LDH@SGQD, and LDH@SGQD‐VP16. f) XPS spectrum, g) high‐resolution C 1s, and h) S 2p of LDH@SGQD and LDH@SGQD‐VP16. i) Photograph under UV light at 365 nm and PL spectra of SGQD, LDH@SGQD, and LDH@SGQD‐VP16.

**Figure** **2**a–c shows the TEM characterization of LDH and LDH@SGQD. The structures of LDH and LDH@SGQD were both hexagonal, the SEM characterization shows the same conclusion (Figure S1, Supporting Information). However, in contrast to LDH, there are many small black points on the surface of LDH@SGQD, which are the SGQDs present on the surface of LDH through electrostatic binding, as shown in the inset Figure 2b. The particle sizes of SGQD are ≈3.0–5.0 nm, and the distribution of SGQDs on the surface of the LDH is even without agglomeration. The lattice structure of SGQDs can be clearly observed by HRTEM in Figure 2c. The results of the dynamic light scattering showed that the size of most particles of LDH@SGQD was ≈100 nm and ≈30 nm smaller than that of the LDH@SGQD‐VP16 particles (Figure 2d). To investigate the thickness changes of LDH@SGQD materials before and after VP16 loading, an atomic force microscope was used (Figure S2, Supporting Information). The thickness of LDH@SGQD (Figure S2a, Supporting Information) was found to be 33.73 nm through the layer height analysis, whereas that of LDH@SGQD‐VP16 increased to 50.74 nm (Figure S2b, Supporting Information). The loading of VP16 increased the spacing between layers, leading to an increase in the thickness of the composite material, which proves that VP16 loading was successfully carried out. The presence of the sulfur elemental analysis by TEM‐EDX (Figure S3, Supporting Information) and SEM‐EDAX (Figure S4, Supporting Information) also confirmed the successful synthesis of the composite.

The zeta potential detection in Figure S5 in the Supporting Information shows that the potential of LDH is positive at ≈+40 mV, while that of SGQD is negative at ≈‐20 mV. In theory, SGQDs can be combined on the LDH surface via electrostatic binding. The potential of LDH@SGQD confirms the above conjecture, which is ≈+20 mV, proving that LDH and SGQD are indeed bound together by electrostatic action. In addition, the potential of VP16 was almost 0 mV, so the potential of LDH@SGQD‐VP16 is the same as that of LDH@SGQD, around +15 mV.

In terms of FTIR, as shown in Figure S6a in the Supporting Information, the characteristic peaks of LDH at 3450 cm^–1^ and 1647 cm^–1^ correspond to the telescopic and flexural vibration peaks of O─H, respectively, and the 1387 cm^–1^ peak corresponds to the telescopic vibration peak of N─O. In addition to the O─H peaks of SGQD, the characteristic absorption peak of SGQD is the stretching vibration peak of C─O at 1121 cm^–1^, which was not observed for LDH. The characteristic peaks of LDH@SGQD contain both N─O and C─O stretching vibration peaks, indicating that SGQD and LDH were combined to form new composite materials. Figure S6b in the Supporting Information compares the variation of characteristic peaks before and after the VP16 drug loading and shows that the characteristic absorption peaks of LDH@SGQD and VP16 were also included in the characteristic absorption peaks of LDH@SGQD‐VP16, indicating that the drug VP16 was successfully carried on the nanomaterials.

The XRD diagram in Figure 2e shows that the position and intensity of the diffraction peaks of LDH@SGQD are the same as those of LDH, indicating that the addition of SGQD does not affect the crystal structure of the composites. After carrying VP16, the position of the diffraction peak shifted slightly to the left, and the strength of the LDH@SGQD‐VP16 peak decreased significantly, indicating that the crystallinity was decreased and that VP16 had been successfully loaded. According to the Bragg equation, with a decrease in the two‐theta of the (003) diffraction peak, the layer spacing increases (Figure S7, Supporting Information). This indicated VP16 was loaded into the LDH layer.

The combustion characteristics of LDH@SGQD and LDH@SGQD‐VP16 were studied by means of thermogravimetry (Figure S8, Supporting Information). The weight loss before 200 °C is owing to the water between the layers. From 200 to 700 °C, the main reason is the crystallization water of Mg‐OH and Al‐OH. The difference between LDH@SGQD and LDH@SGQD‐VP16 is that the weight loss rate after drug‐loading is higher, being 46.5% and 64.291%. This indicated VP16 was loaded successfully.

The XPS characterizations of LDH@SGQD and LDH@SGQD‐VP16 are shown in Figure 2f–h. In the full spectrum analysis, five elements of the composite can be found: C1s (285 eV), O1s (531 eV), Mg1s (1303 eV), Al2p (74), and S2p (168). After carrying VP16, the proportions of C and O were higher than those before. Since the basic structure of VP16 is composed of two elements, C and O, the extra C and O may have been from the drug. This proves that the drug‐loading system has been successfully constructed. As shown in Figure 2g, C1s peak can be divided into three peaks corresponding to C─C (284.8 eV), C─O (286.8 eV), and C═O (288.8 eV). The C─O intensity of LDH@SGQD‐VP16 was much higher, which also provides evidence for formation of the drug‐loading system. The fine spectrum of S2p in Figure 2h, can also be divided into 168.1 and 169.4 eV peaks, which correspond to SO_3_/2p_3/2_ and SO_3_/2p_1/2_, respectively. As SGQD is the only source of S, it is also proved that SGQDs and LDHs constitute a composite structure. The high‐resolution O 1s, Mg 1s, and Al 2p of LDH@SGQD and LDH@SGQD‐VP16 are similar (Figure S9, Supporting Information).

Figure 2i shows the photoluminescence capability of SGQD, LDH@SGQD, and LDH@SGQD‐VP16. They were found to have good water solubility without agglomeration. Under the UV lamp, SGQD presented a bluish fluorescence, which was consistent with the fluorescence excitation/emission spectrum. The maximum emission peak was ≈480 nm, and the maximum excitation peak was ≈380 nm (Figure S10, Supporting Information). The fluorescence of LDH@SGQD under the UV lamp was cyan, indicating that its emission spectrum had a redshift. The maximum emission peak of LDH@SGQD was ≈560 nm, and the maximum excitation peak was ≈470 nm. After the drug VP16 was applied, the fluorescence spectrum was consistent with that of LDH@SGQD. Compared with SGQD, its fluorescence spectrum also shows a redshift, but the range is lower than that of LDH@SGQD, with the maximum emission peak at ≈540 nm and the maximum excitation peak at ≈440 nm. Theoretically, a redshift of fluorescence is more favorable for biological imaging.^[^ ^46^ ^]^

Figure S11 in the Supporting Information shows the sustained release of LDH@SGQD‐VP16 in PBS with different pH values (4.8, 5.8, and 7.0). The release of VP16 within 24 h showed an increasing trend, indicating that the drug could be released from the compound structure. Approximately 50% of VP16 was released in 3 h, indicating a sudden release, after which the release rate decreased significantly, until the amount released reached 90% within 48 h at pH 4.8. With the increase in pH, the total amount of sustained‐release decreased gradually, indicating that LDH@SGQD‐VP16 has the ability of pH‐sensitive release. The results provide a theoretical basis for acid microenvironment‐sensitive targeting and toxicity reduction.

### 2.2 Fluorescence Imaging Capability In Vitro and In Vivo

**Figure 3:**
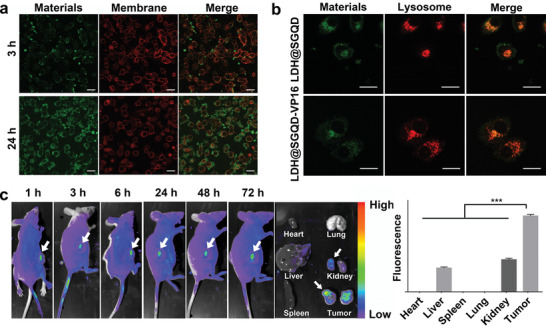
CLSM images of HGC‐27 cells treated with LDH@SGQD‐VP16 (40 mg L^–1^) for 3 and 24 h at 488 nm. a) Cell membrane dyed by Dil at 543 nm. Scale: 20 µm. b) Lysosomal co‐localization of LDH@SGQD and LDH@SGQD‐VP16 at 24 h. Scale: 20 µm. In vivo fluorescence images at 1, 3, 6, 24, 48, and 72 h; organs (heart, liver, spleen, lung, kidney, and tumor) at 72 h, c) post intravenous injection with LDH@SGQD (0.1 mL, 9.2 g L^–1^) visualized at 475 nm and fluorescence quantification using ImageJ. The data in the figures represent the mean ± SD (*n* = 3). Significant differences: ***p < 0.001.

**Figure** **3**a shows the fluorescence imaging of HGC‐27 cells incubated with LDH@SGQD‐VP16 for 24 h at 488 nm excitation light, and cell membrane was stained with Dil at 543 nm. Different from the reported SGQDs,^[^ ^30^ ^]^ LDH@SGQD‐VP16 mainly gathered on the surface of the cell membrane with a small proportion of it entering the cytoplasm. This indicates that the method by which the composite enters cells is different from that of SGQD. Usually, the material released after cell phagocytosis enters lysosomes, in which an acidic environment is conducive to the disintegration of LDH@SGQD‐VP16 to release VP16. The lysosome co‐localization experiment confirmed this hypothesis in HGC‐27 cells (Figure 3b). The lysosomes were marked with a red fluorescent probe, Lyso‐Tracker Red, and the fluorescence of merged images turned orange with the combination of green fluorescence from LDH@SGQD‐VP16, which was transferred to the lysosome after being swallowed. Figure S12 in the Supporting Information shows the fluorescence imaging of LDH@SGQD‐VP16 (80 mg L^–1^) incubated with SGC7901 cells for 1, 6, 12, and 24 h. It was found that the fluorescent signal could be detected on the cells when incubated for 1 h, but the signal was weak and punctuated. With time, the fluorescence intensity of cells becomes stronger. When incubated for 12 h, the outline structure of the cells could be observed, but the boundary was not clear enough. After 24 h, the cell boundaries could be distinguished. This shows that the enrichment of the material in the cell is positively correlated to time. In contrast to HGC‐27 cells, the enrichment of LDH@SGQD‐VP16 in SGC7901 cells was more localized to the periphery of cells, which may affect the therapeutic effect. In summary, the accumulation of LDH@SGQD‐VP16 on the surface of human gastric cancer cells was time‐dependent. This suggests that the concentration of drugs in cells can be determined by detecting the fluorescence intensity of LDH@SGQD‐VP16, which can be used to trace the drug via spontaneous fluorescence, providing a basis for precise visualization therapy.

To investigate the imaging capability in vivo, first, a nude mouse model of HGC‐27 gastric cancer was established. As shown in Figure 3c, 0.1 mL (9.2 g L^–1^) LDH@SGQD was injected into the mouse by intravenous injection. In vivo imaging was performed at 1, 3, 6, 24, 48, and 72 h, and visceral imaging was performed at 72 h, at 475 nm. The results show that fluorescence can be found at the tumor site after 1 h of injection, indicating that LDH@SGQD can target tumor tissue rapidly via blood circulation. With the extension of the injection time, the fluorescence at the tumor site gradually increased, and the retention time at the tumor site was very long. At 72 h after injection, fluorescence emission was still observed. After dissection, fluorescence was observed in the liver, kidney, and tumor, suggesting that LDH@SGQD is metabolized by the liver and kidney. The tumor site can be distinguished by observation, and the material is enriched by, and visualized due to, targeting accumulation and fluorescence intensity. It was demonstrated that LDH@SGQD can be used as a fluorescent probe for tumor visualization therapy.

### 2.3 Safety and Cytotoxicity Analysis

**Figure 4:**
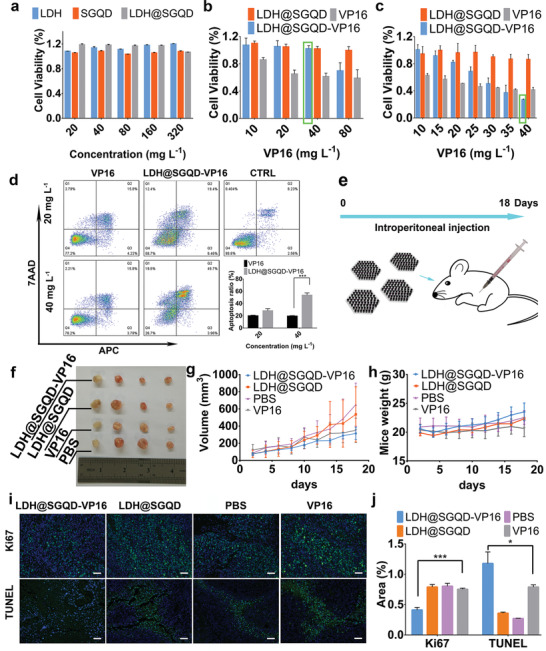
Growth inhibition assay. a) NIH 3T3 cells were incubated with LDH, SGQD, and LDH@SGQD at different concentration (20, 40, 80, 160, and 320 mg L^–1^) (*n* = 4); b) LDH@SGQD‐VP16, LDH@SGQD, and VP16 at different concentration (10, 20, 40, and 80 mg L^–1^) (*n* = 4) ; c) HGC‐27 cells were incubated with LDH@SGQD‐VP16, LDH@SGQD, and VP16 at different concentration (10, 15, 20, 25, 30, 35, and 40 mg L^–1^) (*n* = 4) for 24 h. Cell apoptosis assay by flow cytometry. d) HGC‐27 cells were incubated with LDH@SGQD‐VP16 and VP16 at different concentrations (20 and 40 mg L^–1^) and control for 24 h. e) Scheme of in vivo experiment; f) Photograph of HGC‐27 tumors for each tested group (LDH@SGQD‐VP16, LDH@SGQD, VP16, PBS) at the 18th‐day post intravenous injection; In vivo antigastric carcinoma effect: g) Volume change of tumor and h) weight variety of mice (*n* = 4). i) Cell proliferation analysis (Ki67) and cell apoptosis analysis (TUNEL) of four groups (LDH@SGQD‐VP16, LDH@SGQD, PBS, and VP16) by Ki67/TUNEL (Green), DAPI(Blue). Scale: 100 µm. j) Fluorescence area analysis by ImageJ (*n* = 3). All the data of figures represent the mean ± SD. Significant differences: ***p < 0.001 or *p < 0.05.

**Figure** **4**a shows the safety of the materials using NIH 3T3 cells. Almost all cells survived after incubation with LDH, SGQD, or LDH@SGQD (20, 40, 80, 160 and 320 mg L^–1^) for 24 h, indicating no toxicity to NIH 3T3 cells. Even when the co‐incubation time was extended to 48 h, as shown in Figure S13a in the Supporting Information, the cell viability was still close to 100%. Cytotoxicity analysis was carried out using HGC‐27 and SGC7901 cells. Figure 4b shows the toxicity of LDH@SGQD‐VP16, LDH@SGQD, and VP16 (10, 20, 40 and 80 mg L^–1^) in NIH 3T3 cells for 24 h. At 40 mg L^–1^, the cell viability of the LDH@SGQD‐VP16 was close to 100%, whereas that of the VP16 group was less than 60%. These results indicate that LDH@SGQD can reduce the toxic side effects of VP16 on normal cells. The same result was observed at 48 h (Figure S13b, Supporting Information). When the concentration was increased to 80 mg L^–1^, the cell viability decreased to ≈65%. For safety purposes, the maximum concentration was 40 mg L^–1^ in the HGC‐27 cytotoxicity test. LDH@SGQD‐VP16, LDH@SGQD and VP16 (10, 15, 20, 25, 30, 35 and 40 mg L^–1^) were incubated with HGC‐27 cells for 24 h. At 40 mg L^–1^, the cytotoxicity of LDH@SGQD‐VP16 was ≈1.6 times higher than that of VP16 (Figure 4c). This indicates that LDH@SGQD‐VP16 can increase the cytotoxic effects on HGC‐27, which means that the complex will be suitable for gastric cancer treatment. Similar results were obtained in another gastric cancer cell line, SGC7901, where the cell viability of the LDH@SGQD‐VP16 group was lower than that of the VP16 group at 40 mg L^–1^ concentration, being 14% at 24 h, and 13% at 48 h, respectively (Figure S14, Supporting Information). At 80 mg L^–1^, the survival rate of SGC7901 cells incubated with LDH@SGQD‐VP16 was less than 50%; thus, the concentration of LDH@SGQD‐VP16 in the follow‐up experiment using SGC7901 was set to 80 mg L^–1^. The cytotoxicity test showed that LDH@SGQD‐VP16 had a protective effect on normal cells, and the cytotoxic effects on tumor cells increased. This indicates that LDH@SGQD‐VP16 can serve as a gastric cancer therapeutic agent, with better biocompatibility and higher efficacy compared to existing treatments. Figure 4d shows the apoptosis of HGC‐27 cells treated with VP16 and LDH@SGQD‐VP16 (20 and 40 mg L^–1^, respectively) for 24 h. At 20 mg L^–1^, the apoptotic rates of VP16 and LDH@SGQD‐VP16 were similar, with a difference of only 8%. However, at 40 mg L^–1^, the apoptotic rate of the LDH@SQGD‐VP16 group increased substantially, becoming ≈2.7 times that of the VP16 group. VP16 alone can induce apoptosis in HGC‐27 cells, but the proportion is low and independent of drug concentration. Unlike VP16, LDH@SGQD‐VP16 promoted the apoptosis of HGC‐27 cells in a concentration‐dependent manner. Therefore, LDH@SGQD‐VP16 can improve the therapeutic efficiency of gastric cancer treatment, by promoting apoptosis in gastric cancer cells, as demonstrated in the HGC‐27 cell line.

### 2.4 Therapeutic Effect on Gastric Cancer In Vivo

**Figure 5:**
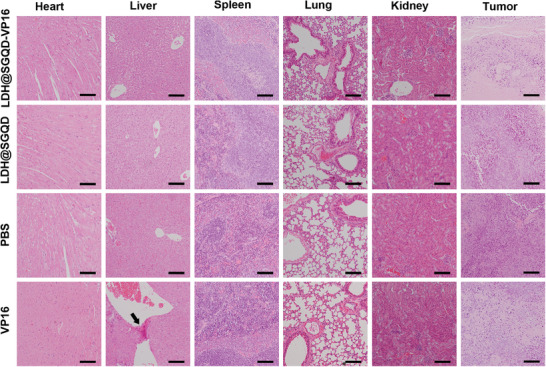
H&E staining images of tumor tissues and other organs (heart, liver, spleen, lung, and kidney) after mice were sacrificed at the 18th‐day post the intraperitoneal injection with various formulas including PBS, VP16, LDH@SGQD, and LDH@SGQD‐VP16. Scale: 100 µm.

To evaluate the therapeutic effect of LDH@SGQD‐VP16 on gastric cancer in vivo, mice were divided into four groups: PBS was the control; the experimental groups were LDH@SGQD, LDH@SGQD‐VP16, and VP16. As shown in Figure 4e, the experiment involved intraperitoneal injection for 18 days, and all the materials were injected every alternate day. The weight of the mice and tumor volume were recorded manually. Figure 4f shows the tumor tissues obtained after the experiment was completed. It can be observed that the tumor masses from the LDH@SGQD‐VP16 group were smaller than those from the other groups. This indicates that the therapeutic effect of VP16 was improved after the nanomaterials were loaded. From the quantitative analysis of the volume size (Figure 4g), it was found that the tumor inhibition rate of LDH@SGQD‐VP16 was higher than that of VP16. This result is consistent with the cytotoxicity test, which means that LDH@SGQD‐VP16 improved tumor treatment. In contrast to VP16, the average weight of the LDH@SGQD‐VP16 group mice continued to increase (Figure 4h). This indicates that the side effects of VP16 are harmful to the body, while LDH@SGQD‐VP16 treatment achieves biosafety. Immunohistochemical analysis of the tumor tissue was performed using paraffin‐embedded sections (Figure 4i). Cell proliferation was detected using the Ki67 labeling method. Green fluorescence indicates cells with positive proliferation, while blue fluorescence indicates nuclei stained by DAPI. Combined with the fluorescence statistical results in Figure 4j, cell proliferation of the LDH@SGQD‐VP16 group was the lowest, 35% less than that of the VP16 group. It has been shown that LDH@SGQD‐VP16 can reduce cell proliferation and inhibit tumor growth. The results of the terminal deoxynucleotidyl transferase dUTP nick‐end labeling (TUNEL) assay showed that the apoptosis rate of the LDH@SGQD‐VP16 group was 35% higher than that of the VP16 group. It has been shown that LDH@SGQD‐VP16 can promote apoptosis, which is consistent with the trend observed in the in vitro apoptosis experiment. Similar results were observed from the hematoxylin and eosin (H&E) staining of tumor tissues (**Figure** **5**). The number of tumor cells in the LDH@SGQD‐VP16 group was the lowest, followed by the that of the VP16 group. Serum analysis of liver and kidney function indexes *γ*‐glutamyltransferase (*γ*‐GT), creatinine (CR), and blood urea nitrogen (BUN) were carried out (**Table** **1**). All indices were within a safe range. Tissue safety and pathology analyses were performed by routine tissue H&E staining (Figure 5). In the VP16 group, some of the hepatocytes were stained dark red, indicating that VP16 had done some damage to hepatocytes. However, LDH@SGQD‐VP16 did not cause any significant damage to the organs. These results indicated that biosafety can be improved upon LDH@SGQD‐VP16 usage.

**Table 1:** The liver parameter (*γ*‐GT) and the kidney parameter (CR, BUN) of mice treated with PBS, VP16, LDH@SGQD, and LDH@SGQD‐VP16

Index	*γ*‐GT [U L^–1^]	CR [µmol L^–1^]	BUN [mg dL^–1^]
PBS	5.852	28.608	11.965
LDH@SGQD	4.028	32.694	13.676
LDH@SGQD‐VP16	6.345	36.782	11.79
VP16	7.4	37.804	13.702

## 3 Conclusion

For the visualization therapy of cancer, we designed a trifunctional integrated nanoprobe, LDH@SGQD‐VP16, which combines the fluorescence imaging capability of SGQDs, the tumor‐targeting properties of LDH in an acidic environment, and the enhanced therapeutic effect of the drug VP16. Chemical characterization proved that LDH and SGQD were combined through electrostatic interactions and had a particle size of ≈100 nm. Compared with SGQD, fluorescence emission has a redshift, which is more conducive to fluorescence imaging of cells and animals. VP16 was successfully loaded with a carrying rate of 28.1%, as confirmed by the FTIR and XRD characteristics. The experimental results of in vitro cells and tumor‐bearing mice show that LDH@SGQD‐VP16 can improve the therapeutic effect of VP16 on the cells in vitro, protect normal cells from damage, inhibit tumor growth in vivo, promote apoptosis, and achieve targeted imaging of the tumor tissue. Compared with other complexes of GQDs and LDH, such as N‐GQDs/CoFe2O4/LDH or PANI/N‐GQD/MO/LDH for breast cancer therapy,^[^ ^26^, ^42^ ^]^ LDH@SGQD‐VP16 is much better in biocompatibility, easier to prepare, and has the advantage of fluorescence visualization for tumor location, which is suitable for effect tracking in real‐time. In addition, the fluorescence intensity of a material can be used to represent drug enrichment. Therefore, the system has achieved the integration of tumor detection, tumor treatment and drug tracking. The ability of LDH@SGQD‐VP16 to accumulate in the tumor microenvironment, in addition to its fluorescence imaging characteristics, makes it an ideal biocompatible nanoparticle for diagnostic and therapeutic vectors. This study provides an experimental basis for LDH@SGQD‐VP16 as a new strategy for the visualization therapy of tumors.

## 4 Experimental Section

### Materials

Etoposide (≥98%), NaOH, Na_2_SO_3,_ Mg(NO_3_)_2_·6H_2_O, and Al(NO_3_)_3_·9H_2_O were purchased from Sinopharm Chemical Reagent Co. Ltd. Phosphate‐buffered saline (PBS), DMEM, and fetal bovine serum (FBS) were obtained from Hyclone. CCK8, Dil, Annexin V‐APC/7‐AAD and LysoTracker Red were purchased from Keygen Biotech. Anti‐Ki67 antibody was purchased from Santa Cruz Biotechnology, and TUNEL Apoptosis Assay Kit was purchased from Beyotime Biotechnology. All the materials were used as received.

### Cell Lines and Animals

Fibroblast NIH 3T3 cells, and gastric cancer HGC‐27 and SGC7901 cells were purchased from the National Collection of Authenticated Cell Cultures. Female nude mice (6–8 weeks old) were purchased from Shanghai Sippr‐BK Laboratory Animal Co. Ltd.

### Synthesis of SGQDs

SGQDs were synthesized according to the previous report.^[^ ^30^ ^]^ Briefly, 1,3,6‐trinitropyrene was stirred with Na_2_SO_3_ solution, transferred to an autoclave, and heated at 130 °C for 12 h via an industrial‐scale procedure.

### Preparation of LDH@SGQD‐VP16

800 mL of deionized water (ddH_2_O) was boiled for 30 min to remove CO_2_. NaOH (0.544 g) was placed in a bottle with ddH_2_O (80 mL) and then placed in a water bath at 60 °C, while stirring with nitrogen (N_2_) at a speed of 400 rpm. After continuous stirring for 5 min, SGQD (1 mg) and VP16 (600 mg) powder were added. Mg(NO_3_)_2_·6H_2_O (1.538 g) and Al(NO_3_)_3_·9H_2_O (0.75 g) were added to ddH_2_O (20 mL); this solution was added to the NaOH solution, drop‐wise, and the mixture was stirred for 3 h. After the reaction, the pellet was collected after centrifugating the mixture at 7000 rpm for 15 min; then, the pellet was dispersed in 70 mL ddH_2_O and placed in a reactor at a constant temperature of 100 °C for 20 h. After the reaction was complete, LHD@SGQD or LHD@SGQD‐VP16 was washed three times to remove impurities, and preserved at 4 °C. The SGQD content and drug‐loading rate were determined by UV‐analysis at 375 and 285 nm, respectively.

### Characterization of LDH@SGQD and LDH@SGQD‐VP16

The size, morphology, and elemental analysis were investigated using Transmission electron microscopy (TEM, JEM 2010F, JEOL Ltd., Japan), scanning electron microscopy (SEM, Sigma 300, Zeiss, GER) and atomic force microscopy (AFM, SPM‐9600, Shimadzu, Japan). The Zeta‐potential values, as well as the hydrodynamic particle sizes were recorded using dynamic light scattering (DLS, Nano‐HT, Zetasizer, Malvern Panalytical, Malvern, UK). X‐ray diffraction (XRD, D/max 2550, Rigaku, GER) patterns were obtained using an X‐ray diffractometer with Cu/K*α* radiation. FTIR was used to detect changes in functional groups using FTIR spectrometer (FTIR, FTS165, Bio‐Rad, USA). UV absorption and fluorescence spectroscopy were performed using a spectrophotometer (UV, 3100, Hitachi, Japan) and a fluorescence spectrophotometer (7000, Hitachi, Japan) respectively. An X‐ray photoelectron spectrometer (XPS, AXIS ULTRA DLD, Kratos, UK) was used to collect the XPS spectra. Thermogravimetric analysis was obtained using a thermal Gravimetric Analyzer (TG, TGA5500, TA, USA).

### Sustained Release of LDH@SGQD‐VP16

LDH@SGQD‐VP16 (VP16: 15 mg) was dispersed in ddH_2_O (3 mL). One milliliter of the solution was placed in a 3.5 KD dialysis bag. Three bottles of 250 mL of PBS with different pH values (pH 4.8, pH 5.8, and pH 7.0) were prepared. The samples were divided into three groups, and the dialysis bags were placed in different bottles in a constant temperature shaker at a speed of 150 rpm and a temperature of 37 °C. The absorbance of VP16 in aqueous solution was detected at 0, 5, 15, and 45 min and at 2, 3, 5, 7, 24, and 48 h using a microplate reader (SpectraMax M5, Molecular Devices, USA) and the release amount was calculated using a standard curve.

### In Vitro and In Vivo Optical Imaging

Approximately 2×10^5^ cells were seeded in a glass‐bottom dish (Cellvis, Mountain View, CA, USA) with 2 mL of culture medium. After 24 h of culture, the LDH@SGQD‐VP16 (VP16: 40 mg L^–1^) were added into the dish. The cell membrane was stained with Dil for 10 min at 37 °C. Lysosomes were stained with LysoRed for 1 h at 37 °C. After incubation, the cells were examined under a confocal microscope (TCS SP5, Leica, GER) using 488 and 543 nm lasers. To investigate the in vivo optical imaging capacity, a nude mouse model of HGC‐27 gastric cancer was established (Female, 6–8 weeks old) and intravenously injected with LDH@SGQD (0.1 mL, 9.2 g L^–1^) through the tail vein. At 1, 3, 6, 24, 48, and 72 h after injection, and organs (heart, liver, spleen, lung, kidney, and tumor) was collected at 72 h, an in vivo imaging system (NightOWL LB983, Berthold, GER) was used to get the results at 475 nm. The fluorescence quantification was obtained using ImageJ (*n* = 3). All experiments were performed in compliance with the relevant laws and institutional guidelines and were approved by the Institutional Animal Care and Use Committee at the Shanghai Institute of Materia Medica, Chinese Academy of Sciences, and the Experimental Animal Center of Tongji University (No: TJAB03821101).

### Cytotoxicity Test

NIH 3T3 cells were cultured in DMEM medium with 1% penicillin‐streptomycin and 10% fetal bovine serum (FBS, Hyclone, USA) at 37 °C in a Thermo cell incubator with 5% CO_2_. SGC7901 cells were cultured in RPMI‐1640 medium supplemented with 1% penicillin‐streptomycin and 10% fetal bovine serum. HGC‐27 cells were cultured in RPMI‐1640 medium supplemented with 1% penicillin‐streptomycin and 20% fetal bovine serum. The CCK8 assay was used to estimate cytotoxicity. Briefly, cells were seeded in 96‐well plates, and ≈5000 cells were seeded in each well (*n* = 4). Different concentrations of materials were added to each group. After 24 and 48 h in culture, CCK8 was added and incubated at 37 °C for 2 h. A microplate reader (ELX800, BIO‐TEK, USA) was used to obtain the experimental data. HGC‐27 cells were cultured in 6‐well plates with 2 mL culture medium for apoptosis analysis. After incubation with LDH@SGQD‐VP16, VP16, and PBS (VP16 concentration of 20 and 40 mg L^–1^) for 24 h, V‐APC and 7‐AAD were used for staining, and the results were obtained using a flow cytometry (FCM, FACS Aria II, BD, USA).

### Treatment of Tumor‐Bearing Mice

A model of the HGC‐27 (1 × 10^6^ cells per mouse, 0.1 mL of saline) subcutaneous tumor was established (Female, 6–8 weeks old). After 10 days, upon tumor growth to a volume of about ≈100 mm^3^, a tumor‐bearing model was established. According to the standard VP16 injection concentration of 15 mg kg^–1^, the corresponding material concentration was allocated, and the PBS containing LDH@SGQD, LDH@SGQD‐VP16, VP16, and PBS were injected intraperitoneally (*n* = 4, Female, 6–8 weeks old). The injection frequency was 2 days, and injections were administered for 18 days; the weight and tumor volume were recorded.

### Immunohistochemical Analysis of Mice

The viscera (heart, liver, spleen, lung, and kidney) and HGC‐27 gastric cancer tissues of the mice were soaked in 4% paraformaldehyde, embedded in paraffin and sliced to a thickness of ≈3 µm. Conventional hematoxylin–Iran Hematoxylin and Eosin (H&E staining) were used to perform routine histopathological analysis. Anti‐Ki67 antibody staining was used for Ki67 proliferation detection using the following steps: incubation at 4 °C overnight, followed by washing with PBS, incubation with the secondary antibody (green fluorescence) for 2 h, re‐staining with DAPI, washing with PBS, and sealing with glycerol. TUNEL apoptosis was detected using a TUNEL Apoptosis Assay Kit. Each section was dripped with 50 µL of detection liquid, incubated at 37 °C for 60 min, and washed with PBS. Serum analysis of liver and kidney function indexes *γ*‐glutamyltransferase (*γ*‐GT), creatinine (CR), and blood urea nitrogen (BUN) were carried out using a biochemical analyzer (Chemray 240, Rayto, China).

### Statistical Analyses

Baseline calibration and curve translation of the data of XRD was adjusted using Jade 5. The data of PL/PLE was normalized and plotted using OriginPro 2017. Baseline calibration of the data of FTIR was adjusted using Omnic 7.3. All of the data were presented as the mean ± standard deviation (SD). Statistical analysis of fluorescence quantification of in vivo fluorescence images was performed using Analysis of variance (ANOVA), and others was performed using Student's *t*‐test using GraphPad Prism 7 software (*p < 0.05, **p < 0.01, and ***p < 0.001).

## Conflict of Interest

The authors declare no conflict of interest.

## Supporting information

Supporting Information

## Data Availability

The data that support the findings of this study are openly available in Dryad at https://doi.org/10.5061/dryad.b2rbnzsfp.
