# Supplementary material for: Trifunctional Graphene Quantum Dot@LDH Integrated Nanoprobes for Visualization Therapy of Gastric Cancer
Source: Adv Healthc Mater. 2021 Jun 10;10(16):2100512. doi: 10.1002/adhm.202100512 (PMC11469055; doi:10.1002/adhm.202100512)
Supplement: Supplementary file 1 — Supporting Information [file ADHM-10-2100512-s001.pdf]

© 2021 Wiley-VCH GmbH

ADVANCED HEALTHCARE MATERIALS

Supporting Information

for *Adv. Healthcare Mater.*, DOI: 10.1002/adhm.202100512

Trifunctional graphene quantum dot @ LDH integrated nanoprobes for visualization therapy of gastric cancer

Bin Wu, Kun Li, Feiyue Sun, Jintong Niu, Rongrong Zhu, Yechang Qian and Shilong Wang**

Supporting Information

Trifunctional graphene quantum dot @ LDH integrated nanoprobes for visualization therapy of gastric cancer

Bin Wu, Kun Li, Feiyue Sun, Jintong Niu, Rongrong Zhu, Yechang Qian and Shilong Wang**

List of contents

Figure S1. SEM images of LDH@SGQD (a) and LDH@SGQD-VP16 (b).
Figure S2. AFM images and height profile along the line A–B of LDH@SGQD (a), and LDH@SGQD-VP16 (b).
Figure S3. TEM-EDX of LDH@SGQD.
Figure S4. EDAX mapping of LDH@SGQD (a) and LDH@SGQD-VP16 (b). (Scale: 5 μm).
Figure S5. Zeta potential of LDH (a), SGQD (b), LDH@SGQD (c), VP16 (d), and LDH@SGQD-VP16 (e).
Figure S6. FTIR spectra of LDH, SGQD, and LDH@SGQD (a); LDH@SGQD, LDH@SGQD-VP16, and VP16 (b).
Figure S7. XRD analysis and the layer spacing calculation of (003) diffraction peak of LDH, LDH@SGQD, and LDH@SGQD-VP16.
Figure S8. TG analysis of LDH@SGQD and LDH@SGQD-VP16.
Figure S9. XPS spectrum. High-resolution O 1s (a), Mg 1s (b), and Al 2p (c) of LDH@SGQD and LDH@SGQD-VP16.
Figure S10. PLE spectra of SGQD, LDH@SGQD, and LDH@SGQD-VP16.
Figure S11. The sustained-release curve of LDH@GQD-VP16 in PBS of different pH values (pH 4.8, pH 5.8, and pH 7.0).
Figure S12. CLSM images of SGC7901 cells treated with LDH@SGQD-VP16 (80 mg L^{-1}) for 1 h, 6 h, 12 h and 24 h at 405 nm, 488 nm, and bright field. Scale: 20 μm.
Figure S13. Growth inhibition assay. NIH 3T3 cells were incubated with LDH, SGQD and LDH@SGQD at different concentration (20 mg L^{-1}, 40 mg L^{-1}, 80 mg L^{-1}, 160 mg L^{-1}, and 320 mg L^{-1}) (a); LDH@SGQD-VP16, LDH@SGQD, and VP16 at different concentration (10 mg L^{-1}, 20 mg L^{-1}, 40 mg L^{-1}, and 80 mg L^{-1}) for 48 h (b). The data in the figures represent the mean ± SD (n = 4).
Figure S14. Growth inhibition assay. SGC7901 cells were incubated with LDH@SGQD-VP16, LDH@SGQD, and VP16 at different concentration (10 mg L^{-1}, 20 mg L^{-1}, 40 mg L^{-1}, and 80 mg L^{-1}) for 24h (a) and 48h (b). The data in the figures represent the mean ± SD (n = 4).

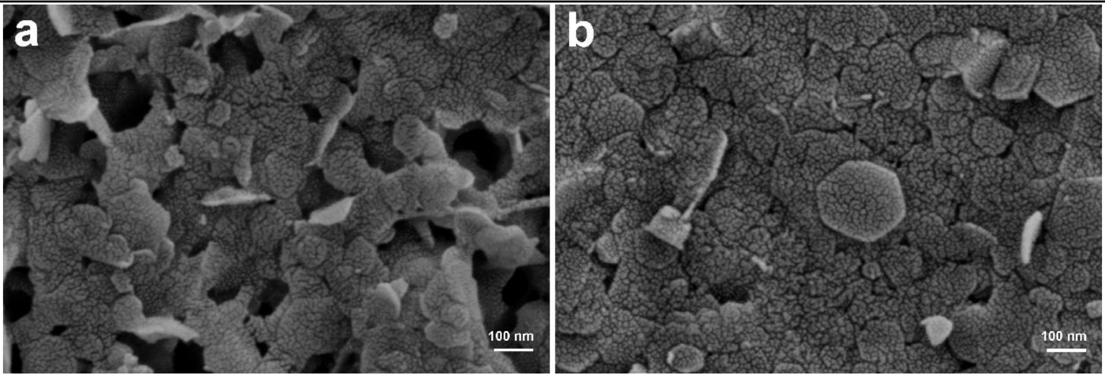

Figure S1. SEM images of LDH@SGQD (a) and LDH@SGQD-VP16 (b).

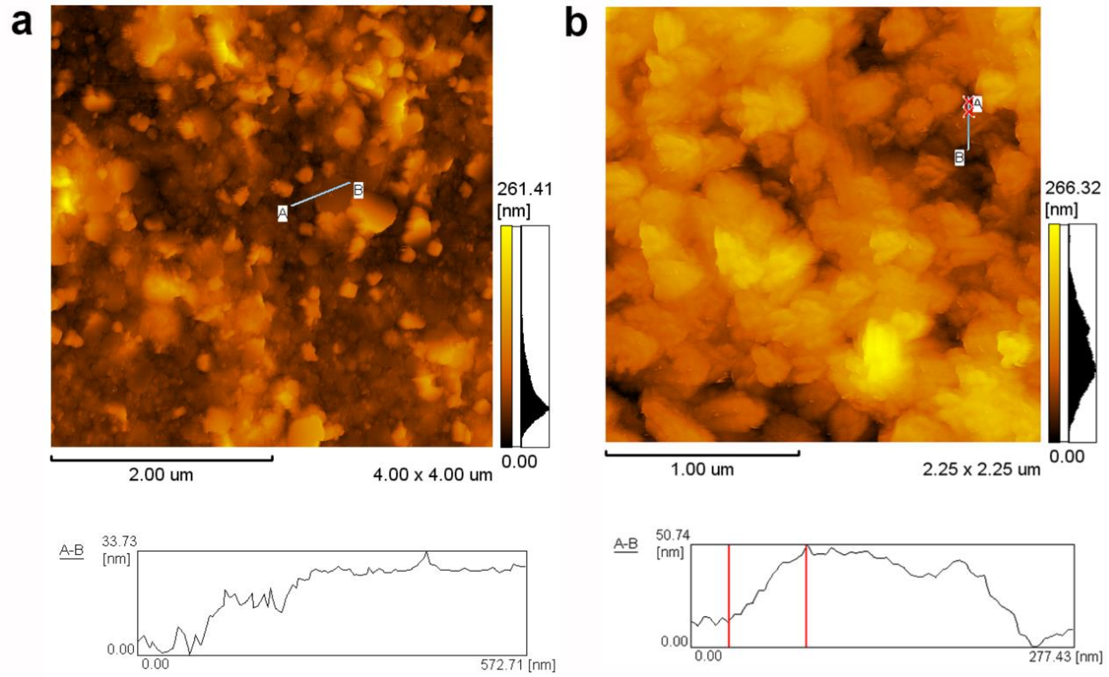

Figure S2. AFM images and height profile along the line A–B of LDH@SGQD (a) and LDH@SGQD-VP16 (b).

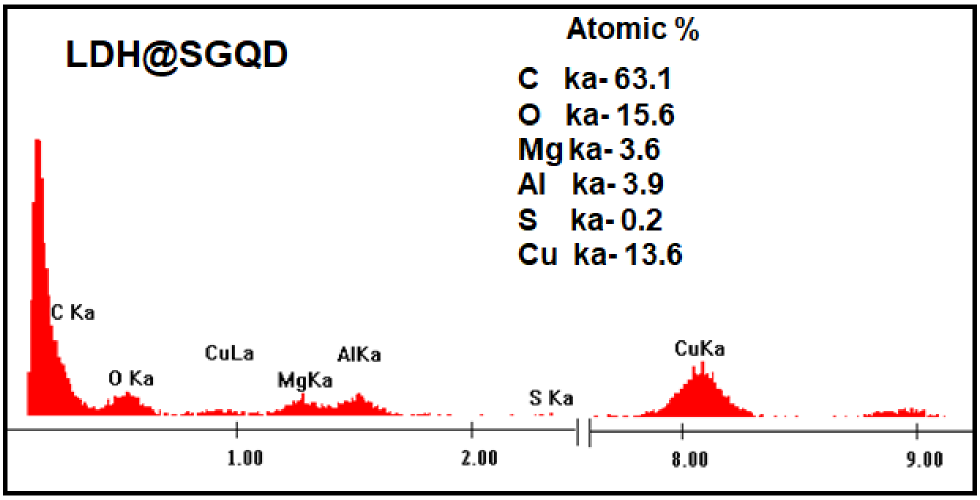

Figure S3. TEM-EDX of LDH@SGQD.

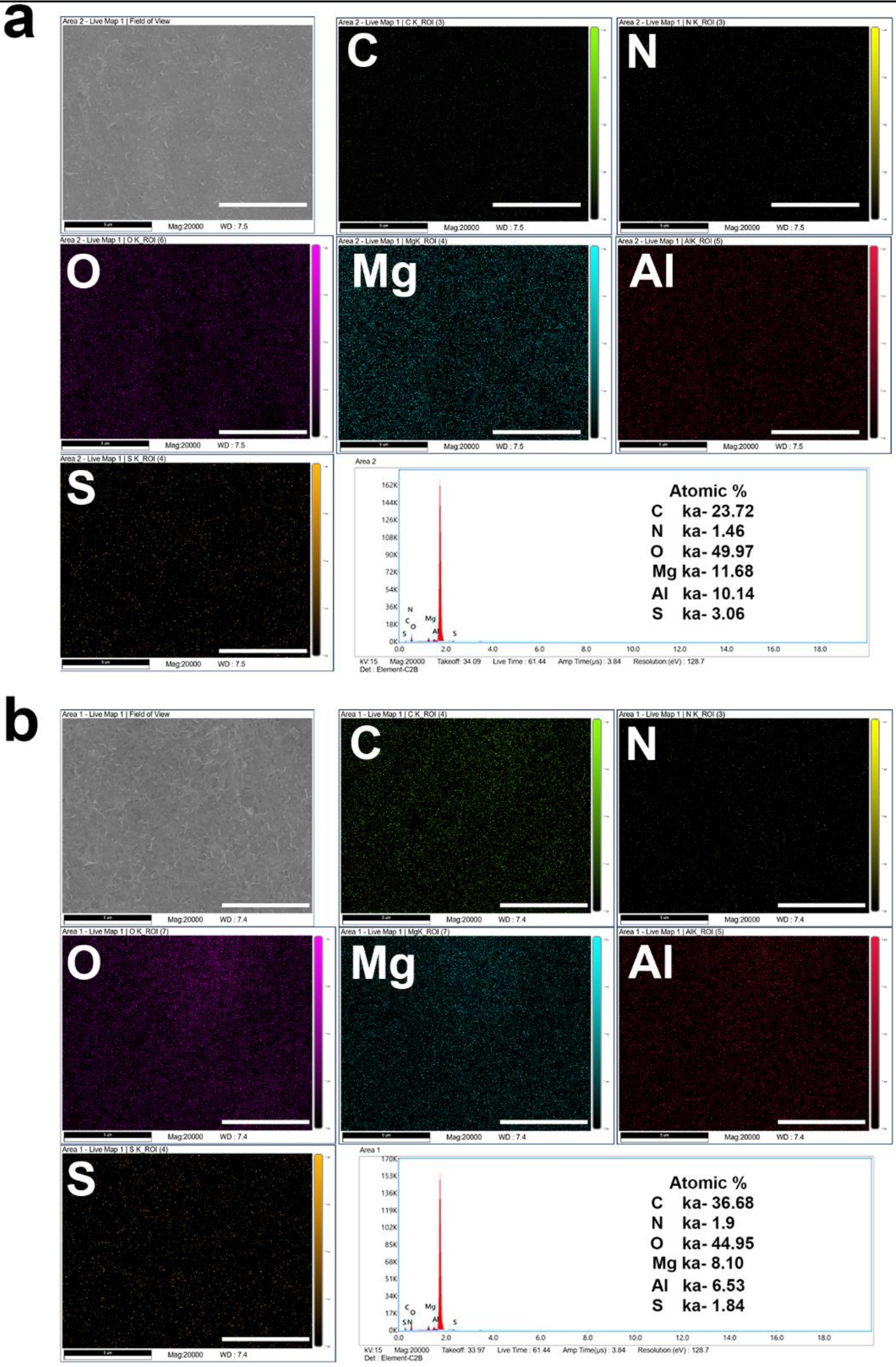

Figure S4. EDAX mapping of LDH@SGQD (a) and LDH@SGQD-VP16 (b). Scale: 5 μm.

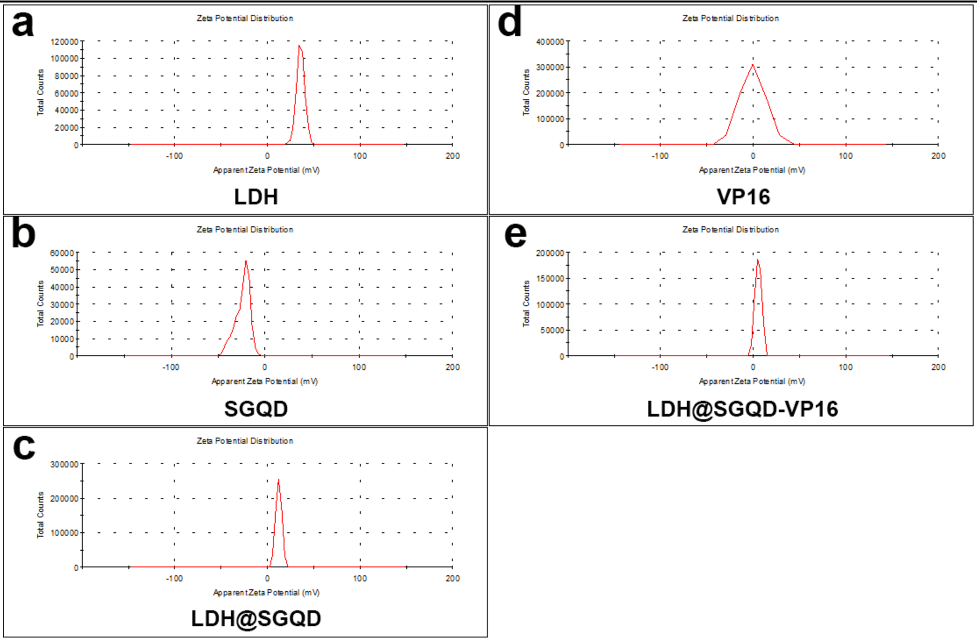

Figure S5. Zeta potential of LDH (a), SGQD (b), LDH@SGQD (c), VP16 (d), and LDH@SGQD-VP16 (e).

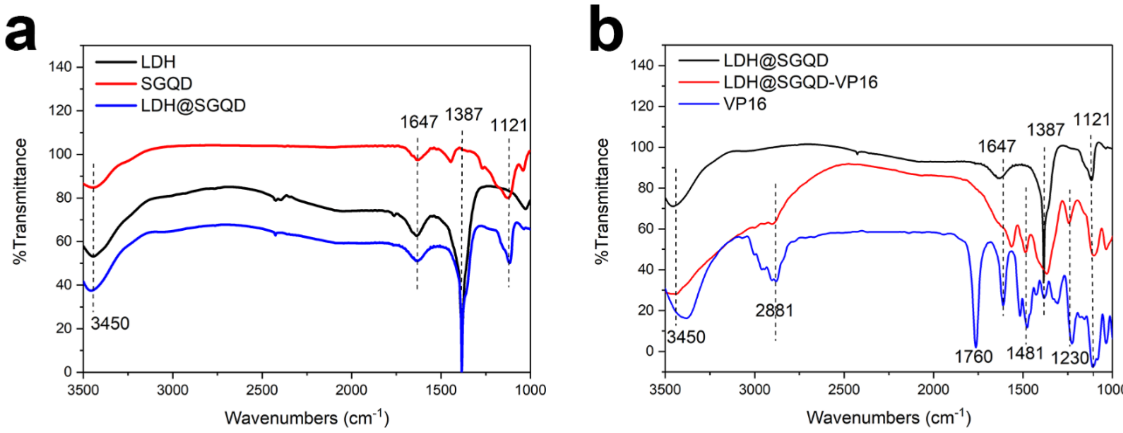

Figure S6. FTIR spectra of LDH, SGQD, and LDH@SGQD (a); LDH@SGQD, LDH@SGQD-VP16, and VP16.

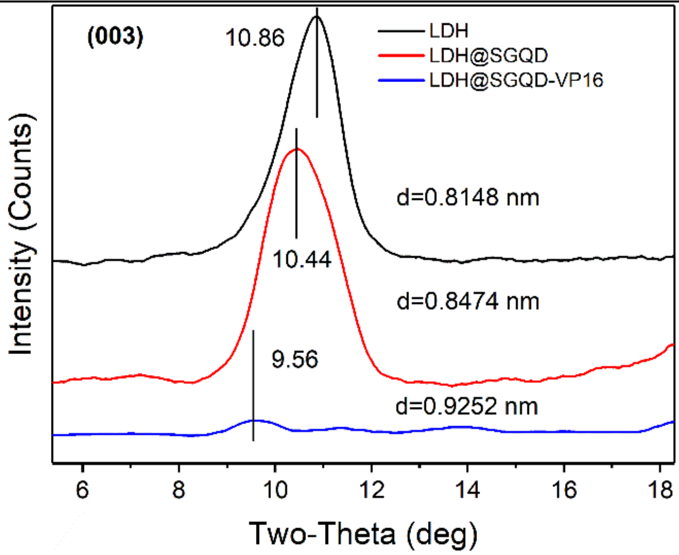

Figure S7. XRD analysis and the layer spacing calculation of (003) diffraction peak of LDH, LDH@SGQD, and LDH@SGQD-VP16.

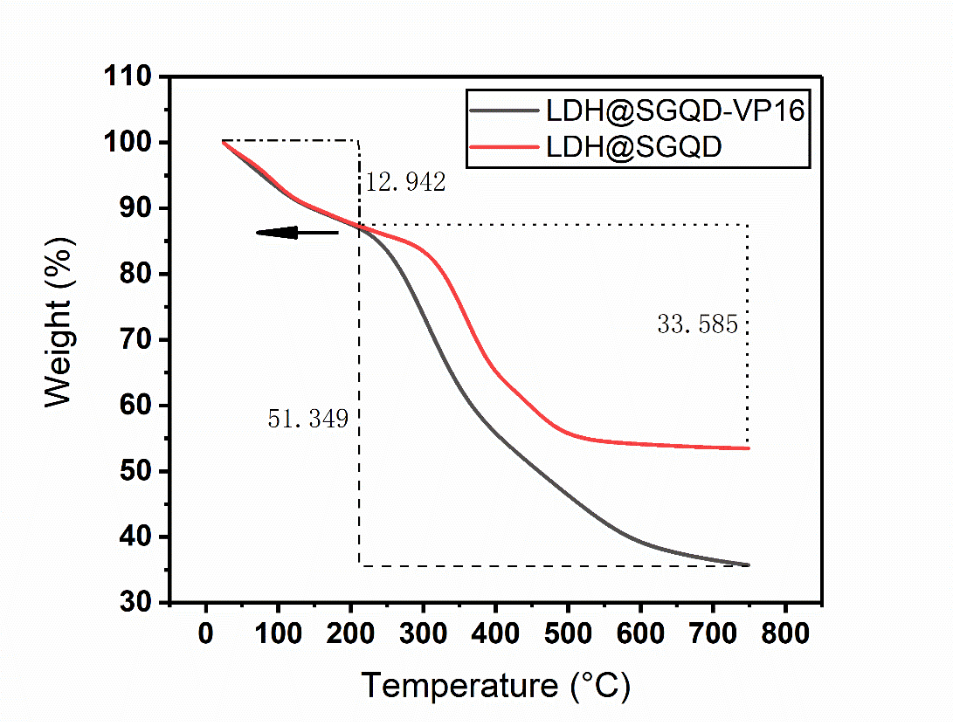

Figure S8. TG analysis of LDH@SGQD and LDH@SGQD-VP16.

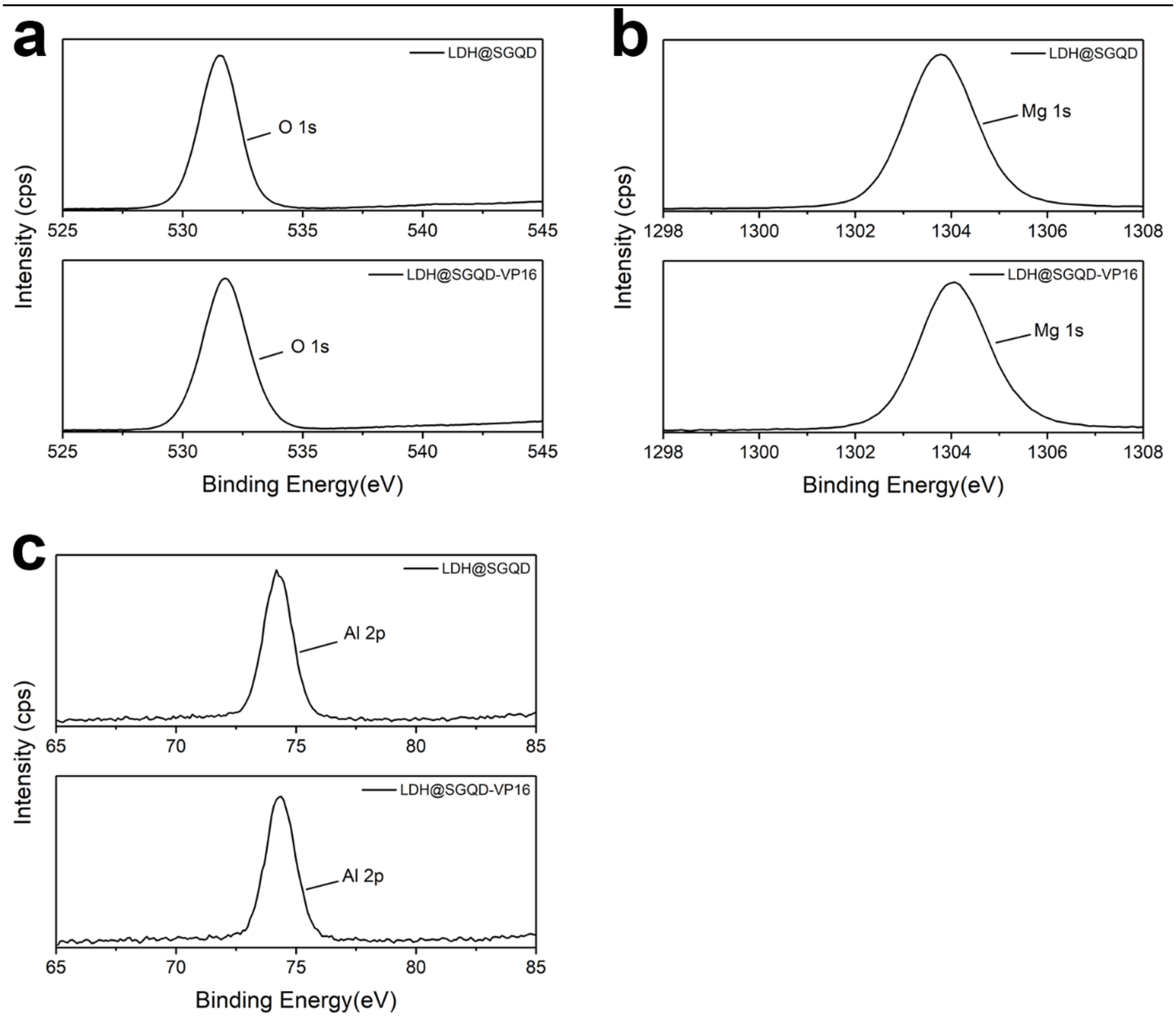

Figure S9. XPS spectrum. High-resolution O 1s (a), Mg 1s (b), and Al 2p (c) of LDH@SGQD and LDH@SGQD-VP16.

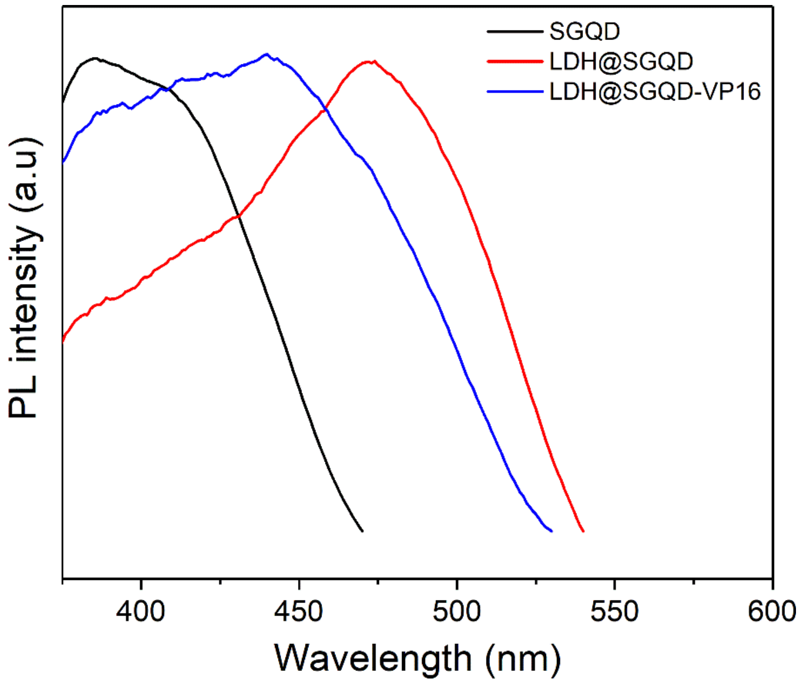

Figure S10. PLE spectra of SGQD, LDH@SGQD, and LDH@SGQD-VP16.

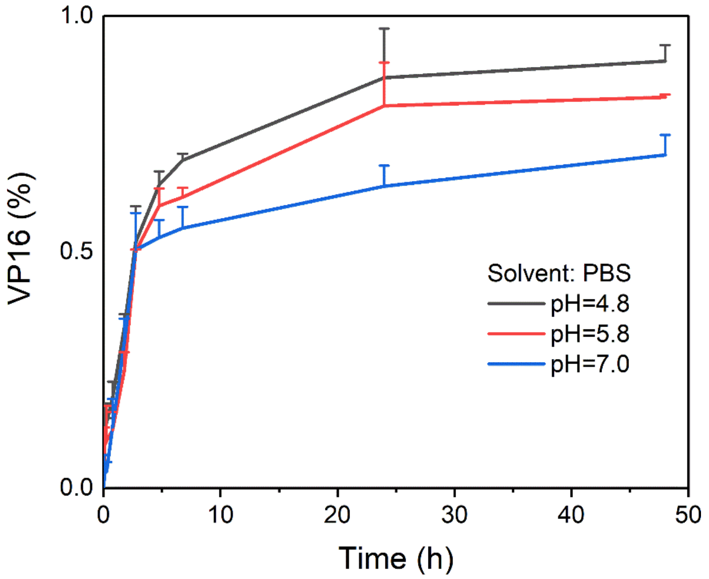

Figure S11. The sustained-release curve of LDH@GQD-VP16 in PBS of different pH values (pH 4.8, pH 5.8, and pH 7.0).

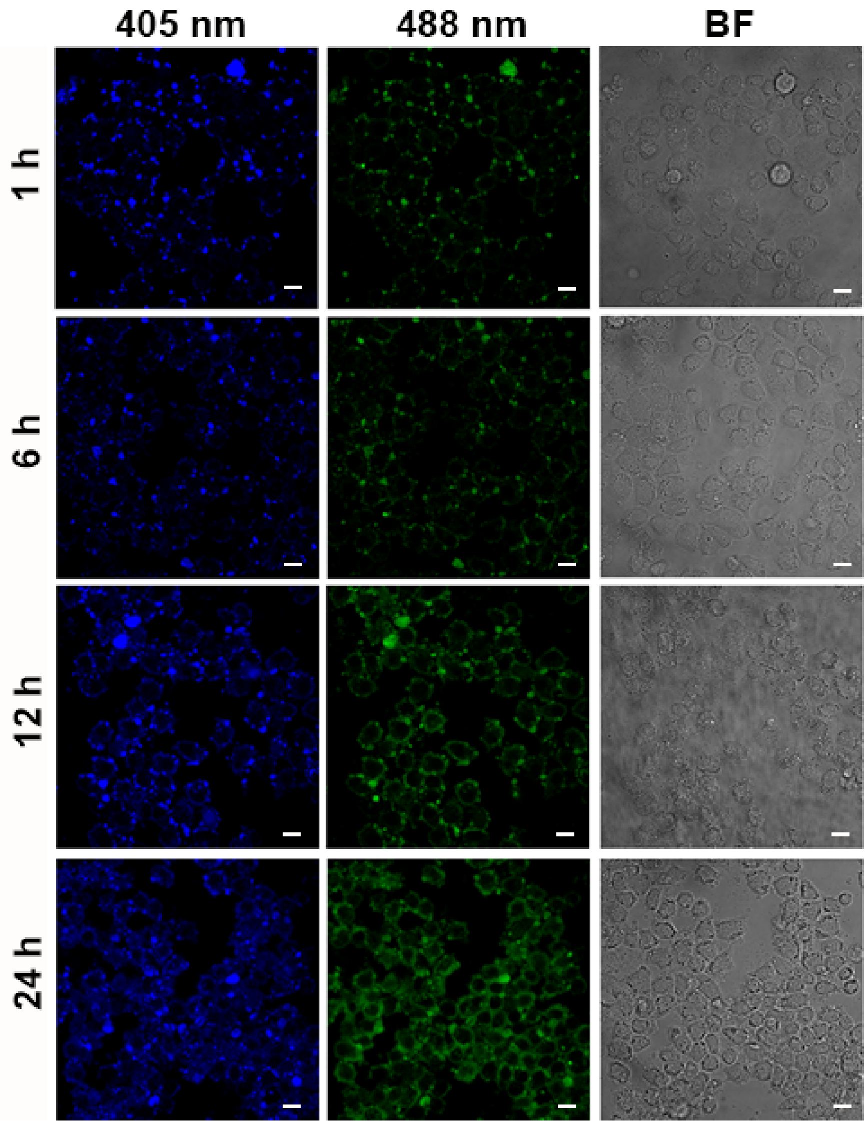

Figure S12. CLSM images of SGC7901 cells treated with LDH@SGQD-VP16 (80 mg L^{-1}) for 1 h, 6 h, 12 h, and 24 h at 405 nm, 488 nm and bright field. Scale: 20 μm.

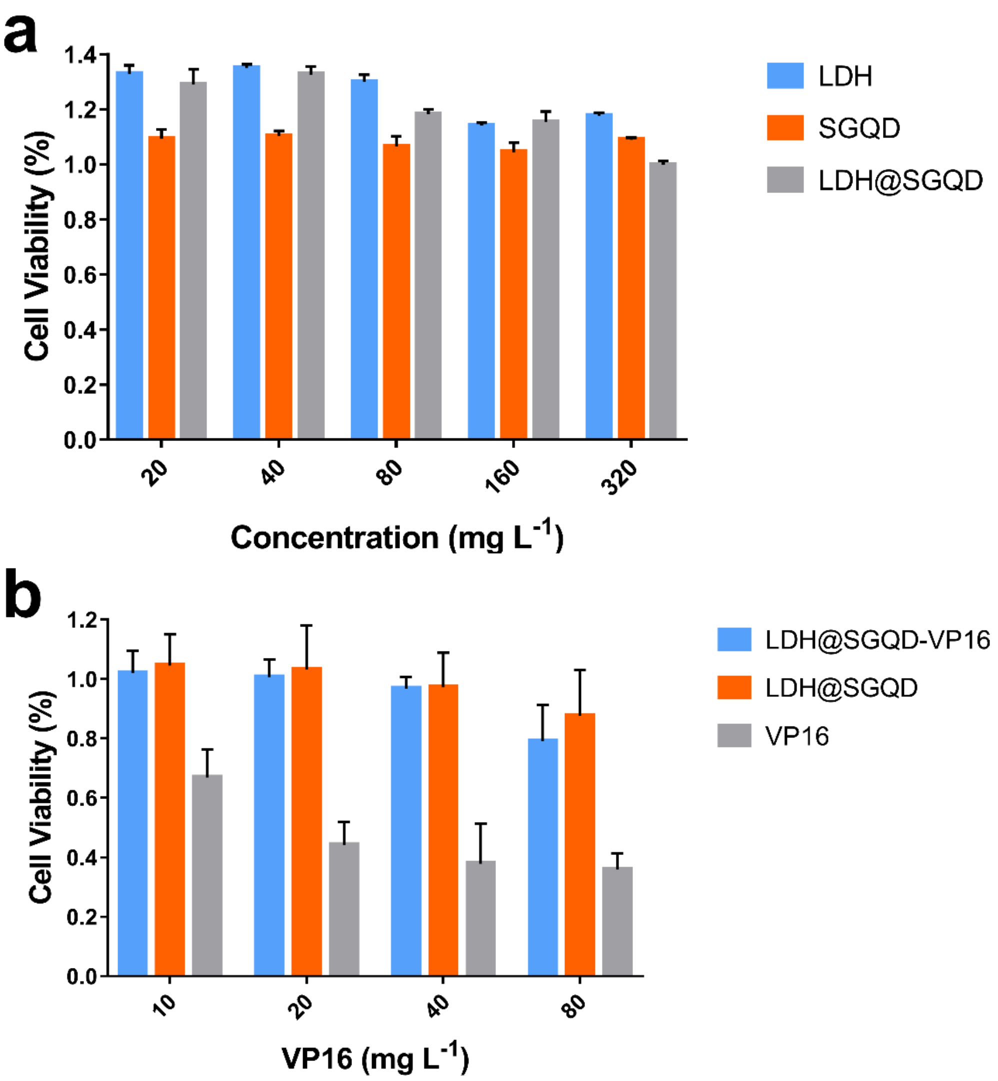

Figure S13. Growth inhibition assay. NIH 3T3 cells were incubated with LDH, SGQD, and LDH@SGQD at different concentration (20 mg L^{-1}, 40 mg L^{-1}, 80 mg L^{-1}, 160 mg L^{-1}, and 320 mg L^{-1}) (a); LDH@SGQD-VP16, LDH@SGQD and VP16 at different concentration (10 mg L^{-1}, 20 mg L^{-1}, 40 mg L^{-1}, and 80 mg L^{-1}) for 48 h (b). The data in the figures represent the mean ± SD (n = 4).

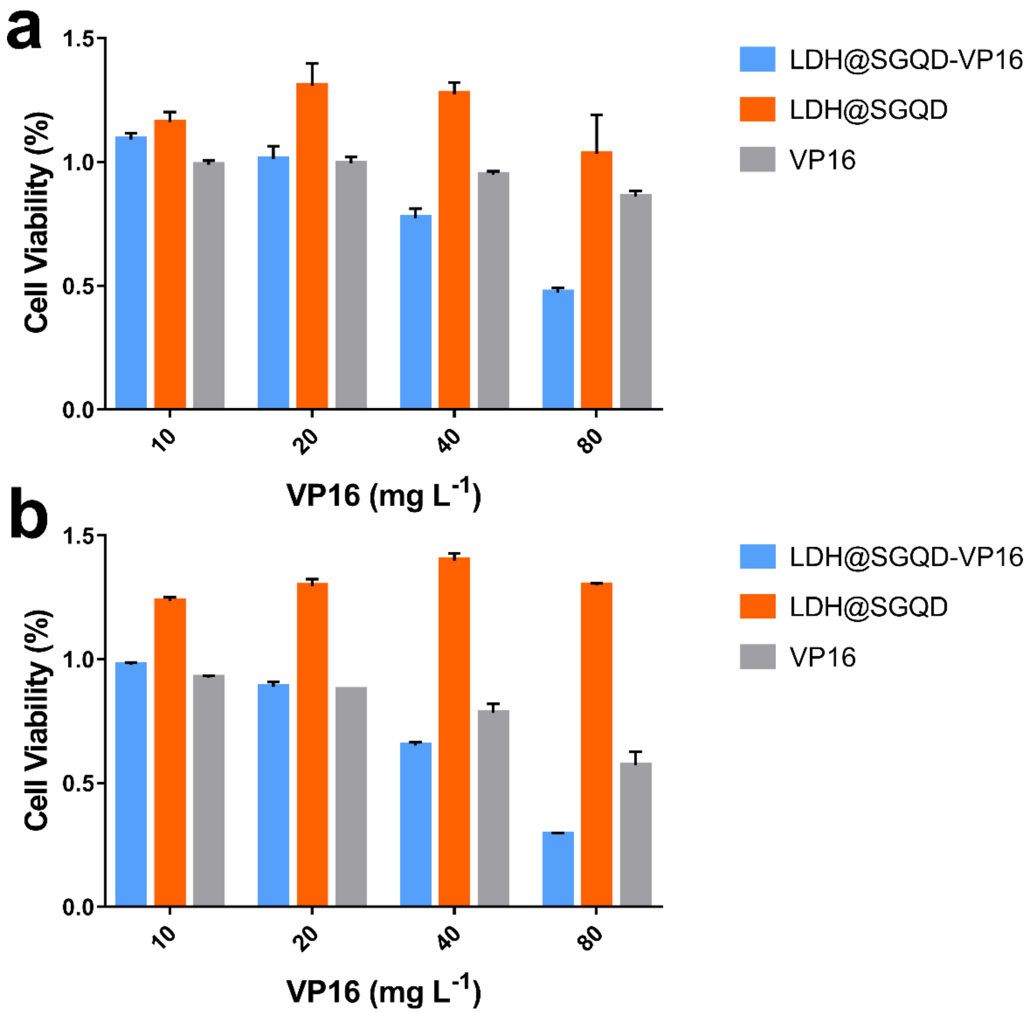

Figure S14. Growth inhibition assay. SGC7901 cells were incubated with LDH@SGQD-VP16, LDH@SGQD, and VP16 at different concentration (10 mg L^{-1}, 20 mg L^{-1}, 40 mg L^{-1}, and 80 mg L^{-1}) for 24h (a) and 48h (b). The data in the figures represent the mean ± SD (n = 4).
